# Development of Restricted and Repetitive Behaviors from 2–19: Stability and Change in Repetitive Sensorimotor, Insistence on Sameness, and Verbal Behaviors in a Longitudinal Study of Autism

**DOI:** 10.1007/s10803-024-06307-4

**Published:** 2024-05-14

**Authors:** Nina Masjedi, Elaine B. Clarke, Catherine Lord

**Affiliations:** 1 Department of Psychiatry and Biobehavioral Sciences, University of California, Los Angeles, Los Angeles, CA 90024 USA; 2 Semel Institute for Neuroscience and Human Behavior, University of California, Los Angeles, 760 Westwood Plaza 68-217, Los Angeles, CA 90024 USA

**Keywords:** RRBs, Autism, Factor analysis, Growth modeling, Developmental trajectories, Insistence on sameness

## Abstract

This study examined trajectories of repetitive sensorimotor (RSM), insistence on sameness (IS), and verbal RRBs from ages 2–19 in a well-characterized longitudinal cohort. We also tested the factor structure of the ADI-R restricted and repetitive behavior (RRB) domain at age 19 and the inclusion of a verbal RRBs factor, in addition to previously identified RSM and IS factors, at ages 9 and 19. The ADI-R was administered to 193 participants at five timepoints from 2 to 19. Confirmatory factor analysis (CFA) was used to examine the factor structure of ADI-R RRB data. Change in RRB subtypes was examined using group-based trajectory modeling and multilevel modeling. RSM and IS behaviors generally decreased from 2 to 19, with some participants experiencing increases from ages 2–9. 46.46% of participants experienced increasing verbal RRBs from 2 to 9, followed by a plateau from 9 to 19. The remaining participants had few to no verbal RRBs throughout development. Multilevel modeling indicated ADOS CSS scores were associated with change in RSM trajectories only. Child IQ was not associated with change in any RRB trajectories. CFA indicated previously-derived RSM and IS factors fit age 19 ADI-R data well. The inclusion of a third factor, verbal RRBs, worsened goodness-of-fit statistics and was ultimately omitted. Our findings suggest the RRB factor structure of the ADI remains consistent into early adulthood and illustrate developmental continuity and change in RRBs. For autistic individuals with fluent speech, RRB presentation may be influenced by verbal ability in ways that are highly variable, but clinically meaningful.

**Supplementary Information:**

The online version contains supplementary material available at 10.1007/s10803-024-06307-4.

Though autism spectrum disorder (ASD) is considered a lifelong condition, the behavioral phenotype of ASD is not static. In other words, symptoms can change markedly across development. Symptoms of ASD are typically conceptualized under two core categories: social communication and restricted and repetitive behaviors (RRBs). Social communication impairments can include deficits in eye contact, limited facial expressions and gestures, and difficulty maintaining relationships (American Psychiatric Association, 2014). RRBs include stereotyped motor movements, repetitive use of objects, and unusual sensory interests (APA, 2014). Though historically a target of behavioral interventions (Schreibman, 1997), RRBs are not inherently impairing, and many autistic individuals report RRBs can be an important form of self-soothing (Livingston et al., 2019). RRBs vary both across and within individuals. Understanding RRB symptoms, as well as their associations with other phenotypic characteristics such as IQ and social communication impairments, across the life course may facilitate a deeper understanding of the presentation of ASD. This study leverages a well-characterized longitudinal cohort of individuals with ASD identified in early childhood to examine trajectories of RRB subtypes from ages 2 to 19. In accordance with mixed preferences, we refer to this sample interchangeably as “individuals with autism” and “autistic individuals” throughout.

Prior work in the current sample examined trajectories of social communication deficits over the same period, from ages 2 to 19, and found significant improvements in social communication with increasing age (Bal et al., 2019). Social communication improved in participants of all verbal abilities, though verbally fluent participants experienced greater improvements in social communication than minimally-verbal participants and participants with delayed speech (Bal et al., 2019). Another study of this cohort examined trajectories of RRBs, specifically Repetitive Sensory Motor (RSM) and Insistence on Sameness (IS) behaviors, from ages 2 to 9 (Richler et al., 2010). Richler et al. found three distinct trajectory groups for RSM scores—a mild group, a slightly decreasing group, and a consistently severe group—as well as three groups for IS scores—a mild group, an increasing group, and a moderate group. Whereas RSM scores had consistent severity or improved slightly from ages 2–9, IS scores tended to increase during this period. To extend these previous findings, the current study examines RRB trajectories through early adulthood using the factor structure previously derived by Richler et al. (2010).

Prior studies of the current longitudinal sample used a priori analytic groupings based on participant language level (Bal et al., 2019) and diagnostic status (Richler et al., 2010) to identify trajectories of ASD symptoms. In contrast, this paper uses a data-driven approach to identify participant trajectory groupings. To enhance the clinical utility of this work, we include information on the phenotypic and demographic characteristics in our empirically derived trajectory subgroups.

## Measuring the Underlying Structure of RRBs

Many diagnostic instruments measure RRBs, notably the Autism Diagnostic Interview-Revised (ADI-R; Rutter et al., 2003), the Autism Diagnostic Observation Schedule, 2nd Edition (ADOS-2; Lord et al., 2012), and the Repetitive Behavior Scale-Revised (RBS-R; Bodfish et al., 2000). In line with prior analyses of this sample (Richler et al., 2010), the current study used ADI-R scores to assess developmental change in RRBs. A separate cross-sectional study of the Simons Simplex Collection (SSC; n = 1825), found the ADI-R captured subcategories of RRBs in more detail than the ADOS-2 (Bishop et al., 2013). In contrast to the RBS-R, the ADI-R is a clinical interview rather than a questionnaire and is therefore less influenced by reporting bias (McDermott et al., 2020; Mirenda et al., 2010).

Previous factor analyses of the ADI-R in both this sample at ages 2, 3, 5, and 9, (Richler et al., 2007, 2010) and other independent samples of children with ASD (Bishop et al., 2013) found two emergent factors of RRBs: Repetitive Sensorimotor (RSM) and Insistence on Sameness (IS) behaviors. RSM includes hand and finger mannerisms and complex body mannerisms, repetitive use of objects, and unusual sensory interests. IS includes compulsions and rituals, difficulties with changes in routine, and resistance to trivial changes (Richler et al., 2007, 2010). Separate factor analyses of the ADI-R and the RBS-R have identified similar RSM and IS factors, suggesting these subcategories are not a product of the structure of the ADI-R (Cuccaro et al., 2003). Little existing work has examined the stability of this two-factor structure after late childhood. The current study sought to address this gap by examining the factor structure of the ADI-R in early adulthood, at age 19.

Other factor analyses of the ADI-R have identified additional RRB factors including stereotyped speech (Hiruma et al., 2021) and circumscribed interests (Lam et al., 2008). Thus, the current study also tested the inclusion of a third factor, verbal RRBs, at ages 9 and 19.

### Verbal RRBs

Communicative use of language (i.e., verbal ability) varies amongst people with autism, is a key predictor of developmental trajectories (Magiati et al., 2014; Pickles et al., 2020), and may be important to RRB trajectories. Some RRBs, such as verbal rituals and neologisms/idiosyncratic language, cannot be present in nonverbal autistic individuals. Other RRBs, such as circumscribed interests and unusual preoccupations, may be present in autistic individuals of varying language abilities but more obvious in verbally fluent individuals who can speak about their interests and preoccupations at length. In this study, verbal RRBs are defined as behaviors at least partially reliant on verbal ability, such as verbal rituals and circumscribed interests (see Table 1).

**Table 1:** ADI-R raw items include in RSM, IS, and verbal trajectory sums

Trajectory				ADI-R Item
RRB (Total)	RSM	IS	Verbal	
✓	✓			*Repetitive use of objects*
✓	✓			*Unusual sensory interests*
✓	✓			*Hand and finger mannerisms*
✓	✓			*Other complex mannerisms*
✓		✓		*Resistance to trivial changes in environments*
✓		✓		*Difficulties with changes in routine*
✓		✓		*Compulsions and rituals*
✓			✓	*Verbal rituals*
✓			✓	*Unusual preoccupations*
✓			✓	*Circumscribed interests*
✓			✓	*Stereotyped utterances and delayed echolalia*
✓			✓	*Neologisms/idiosyncratic language*

The items stereotyped utterances and delayed echolalia; and neologisms/idiosyncratic language were not used in the final trajectory sums

Trajectories of verbal RRBs have not been studied in detail (Volden & Lord, 1991). There is some ambiguity as to how to define and classify this subtype of autism symptoms. In the ADI-R, autism is diagnosed via symptoms in three domains: social, RRBs, and communication, with this final domain traditionally including verbal symptoms (Le Couteur et al., 1989). Thus, symptoms such as stereotyped utterances and delayed echolalia and neologisms/idiosyncratic language are included in the communication algorithm of the ADI-R, rather than the RRB algorithm (Rutter et al., 2003). However, the current DSM-5 conceptualization of ASD stratifies symptoms into two domains—social communication and RRBs—with some communication symptoms included in the RRB domain (APA, 2014). Clinically, there can be considerable overlap in the presentation of RRB and communication symptoms, as autistic individuals with fluent speech may be able to express the nature and intensity of their restricted interests and desire for sameness more effectively than minimally- or non-verbal autistic individuals.

This study adds to the existing literature on verbal RRBs in three ways. First, to better understand how verbal symptoms of autism are associated with the underlying structure of RRBs, we tested the inclusion of a verbal factor on the factor structure of the RRB domain of the ADI-R in late childhood (age 9) and early adulthood (age 19). Due to our sample’s relatively late emergence of language fluency (see below) we were unfortunately unable to test the inclusion of a verbal factor at earlier ages. Second, we examined trajectories of verbal RRBs separately from trajectories of RSM and IS behaviors. Finally, for RSM and verbal RRBs specifically, we also compare developmental trajectories based on DSM-5 RRB criteria to those based on ADI-R algorithm criteria.

### Developmental Characterizations of RRBs

Though few studies have examined RRBs from early childhood into early adulthood, prior work in separate samples examined changes in RRBs across childhood. Like Richler et al. (2010), these studies found many RRB behaviors increase from early to late-childhood (Courchesne et al., 2021; Guthrie et al., 2013). In contrast, cross-sectional studies of adults with ASD indicate RSM behaviors decrease with increasing age (Bishop et al., 2013; Evans et al., 2017; Uljarević et al., 2021). Considered together, studies of RRBs in childhood and early adulthood suggest RRB trajectories before and after late childhood may follow differing slopes, with RRBs increasing through mid- to late-childhood and decreasing into adolescence and early adulthood. However, longitudinal data are needed to confirm these patterns. Currently, there is also insufficient evidence to establish whether the slopes of RRB trajectories at different developmental stages vary by RRB subtypes. The present study builds upon existing cross-sectional findings that RRBs increase in childhood and decrease in early adulthood to longitudinally examine change in the slopes of RSM, IS, and verbal trajectories and test whether these slopes significantly differ before and after late-childhood (i.e., age 9).

### Study Aims

Our first aim was to determine whether the previously-identified two-factor structure of ADI-R RRB items was consistent with ADI-R scores in our sample at age 19. In line with the DSM-V conceptualization of autism symptomology, which collapses RRB and communication symptoms, we also wanted to consider whether the inclusion of a verbal RRB factor, in addition to the previously identified RSM and IS factors, fit well with age 9 and 19 ADI-R data. Unfortunately, we were unable to test the utility of including a verbal factor at earlier ages given the timing of when fluent verbal skills emerged for many participants in this sample. We anticipated that the two-factor structure of the ADI-R would remain stable at age 19. Given the lack of prior literature, we had no specific hypotheses about the utility and validity of including a verbal RRBs factor.

Our second aim was to examine trajectories of ADI-R RSM, IS, and verbal scores from ages 2 to 19, and to compare the phenotypic characteristics (e.g., IQ, ADOS CSS) of participants based on their trajectory group inclusion. We expected to replicate prior work by Richler and colleagues in this sample (2010). Given prior findings that social communication symptom trajectories were associated with IQ (Bal et al., 2019), we anticipated IQ and social communication symptom severity would be related to change in RRB symptoms over time.

Our third aim was to investigate differences in the rates of change of the RSM, IS, and verbal trajectory subgroups, respectively, from age 2–19. In line with prior findings in this sample and others, we expect ADI-R RSM scores to decrease and IS scores to increase with increasing age (Bishop et al., 2013; Evans et al., 2017). Given the lack of prior literature, we had no specific hypotheses about verbal RRB trajectories.

## Method

### Participants

193 consecutive referrals to community-based developmental clinics in North Carolina, Chicago, and Michigan who ever received a diagnosis of autism and had at least one ADI-R (*m* = 3.9, SD 1.2) were included in the current study. Given the current study’s focus on a core feature of ASD, restricted and repetitive behaviors, consecutive referrals who never received an ASD diagnosis were excluded from the present analyses. Fifty-eight participants were non-white and 26 were female. Missing data occurred as the result of refusal, change of place of residence, and inaccessible status.

### Procedure

Face-to-face visits occurred at approximately ages 2, 3, 5, 9, and 19. Research-reliable research assistants, trainees, or clinical psychologists administered the ADI-R and other diagnostic measures. Parents and participants over the age of 18 who were their own legal guardians signed a consent form as required by the applicable institutional review board(s) prior to each visit.

#### Measures

*Autism Symptomology****.*** The ADI-R (Rutter et al., 2003) was administered at ages 2, 3, 5, 9, and 19. ADI-R current raw scores were used to calculate trajectories for verbal RRBs, RSM, and IS trajectories (Table 1). To capture nuanced changes in RRB subtypes, the raw score (0 through 3, with 3 meaning most severe) for each item was used in a total sum for the domain. If an item in a domain was missing, it was treated as a zero. If all items in a domain were missing, a score for that domain was not calculated. For verbal RRB trajectory analyses, only participants who received a score of 0 at one or more timepoints on the ADI-R item “Overall Level of Language,”—indicating daily, functional use of language involving phrases of three or more words—were included. At age 2, 7% of participants received a score of 0 for the “Overall Level of Language” item. This increased to 36% at age 3, 60% at age 5, 70% at age 9, and finally 77% at age 19. With the exception of “Unusual Preoccupations,” the percentage of participants whose caregiver endorsed specific verbal symptoms in early childhood was relatively low (Table 6).

*IQ.* At face-to-face assessments, verbal and non-verbal IQ were calculated using one of the following assessments (from most to least difficult): the Wechsler Abbreviated Scale of Intelligence (Wechsler, 1999), the Differential Ability Scales (Elliott, 2007), or the Mullen Scales of Early Learning (Mullen, 1995). Selection of IQ measures in this sample is extensively described in Anderson et al. (2014) and Lord et al. (2006). Childhood IQ was determined from the first available childhood face to face visit (m = 3.32; SD 2.65) and adulthood IQ was determined from the most recent face to face visit (m = 18.91; SD 1.25.)

### Data Analysis

#### Aim 1: Test RSM and IS Factors at Age 19 and Inclusion of a Verbal Factor at Ages 9 and 19

To test whether the previously-identified two factor structure of ADI-R RRB items remained consistent at age 19, and to examine the effects of including an additional factor for verbal RRBs at ages 9 and 19, confirmatory factor analyses were performed using the sem command in Stata 16. Unfortunately, given the limited number of participants with sufficient verbal fluency and/or verbal symptoms at earlier ages (Table 6), we were unable to test the effects of including an additional factor for verbal RRBs at ages 2, 3, and 5. CFA was used because the factor structure of the ADI-R RRB items has previously been validated from early to late childhood in this sample (Richler et al., 2007, 2010) and other independent samples of ASD (Bishop et al., 2013). Due to our a priori theoretical assumption that items included in the additional third factor, Verbal RRBs, would load together, CFA was also used to measure the fit of a three-factor model at age 19 and at age 9 (Bollen et al., 2002; Marsh et al., 2014). The Tucker-Lewis Index (TLI), Comparative Fit Index (CFI), and root mean squared error of approximation (RMSEA) were used to assess model fit. Values closer to “1” using the TLI and CFI and values closer to “0” using the RMSEA indicate better model fit. Recommended cutoffs for well-fitting models are greater than 0.95 for the TLI and CFI and ≤ 0.06 for the RMSEA. Notably, there is some evidence these cutoffs may be overly exclusive in small samples (Hu & Bentler, 1999). To maintain consistency with prior factor analyses of the ADI-R (Richler et al., 2007, 2010), the IS and RSM factors were allowed to covary.

#### Aim 2: Examine RSM, IS, and Verbal Trajectories from 2–19

**Fig. 1:**
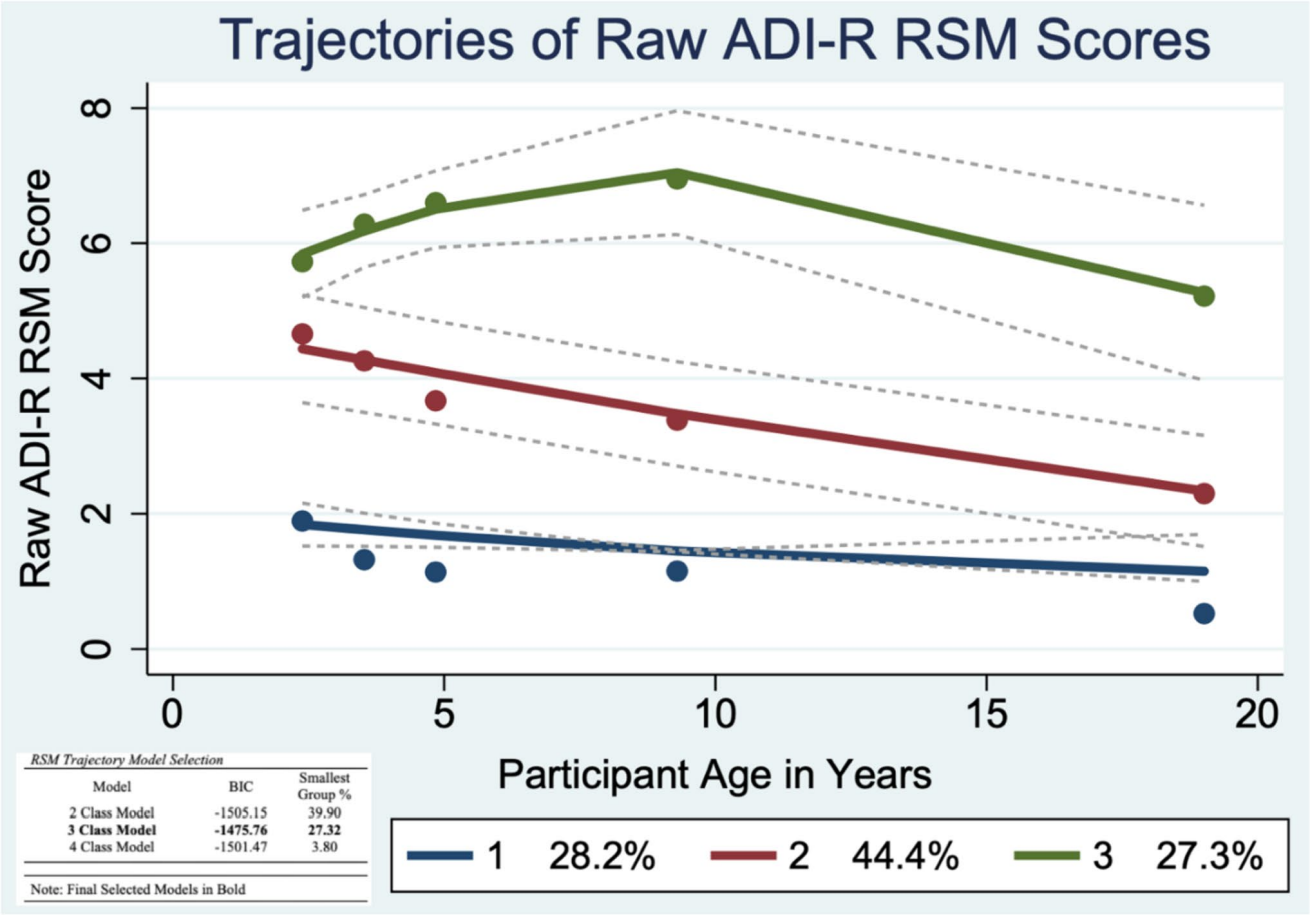
Trajectories of Raw ADI-R Restricted Sensorimotor (RSM) Scores

**Fig. 2:**
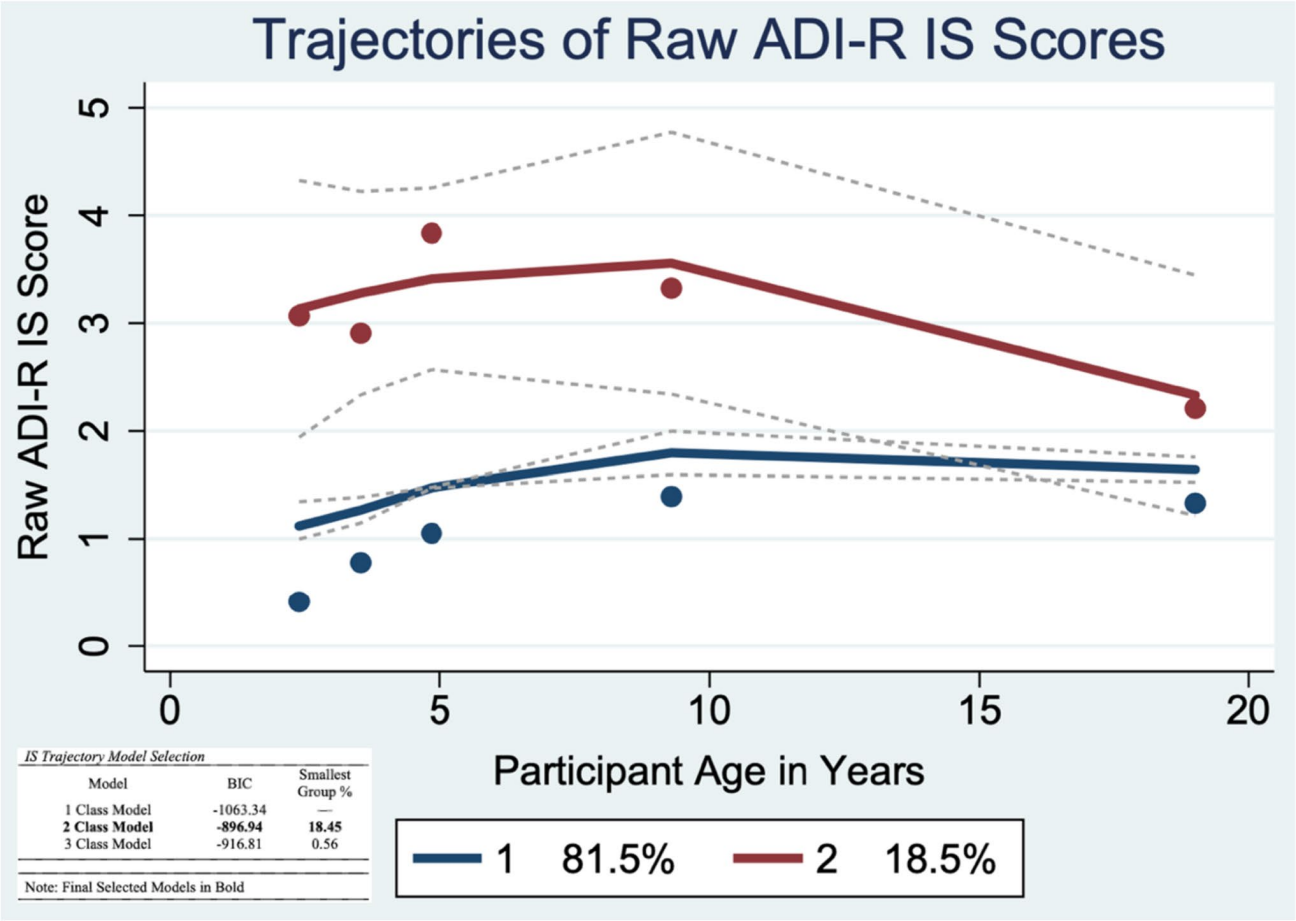
Trajectories of Raw ADI-R Insistence on Sameness (IS) Scores

**Fig. 3:**
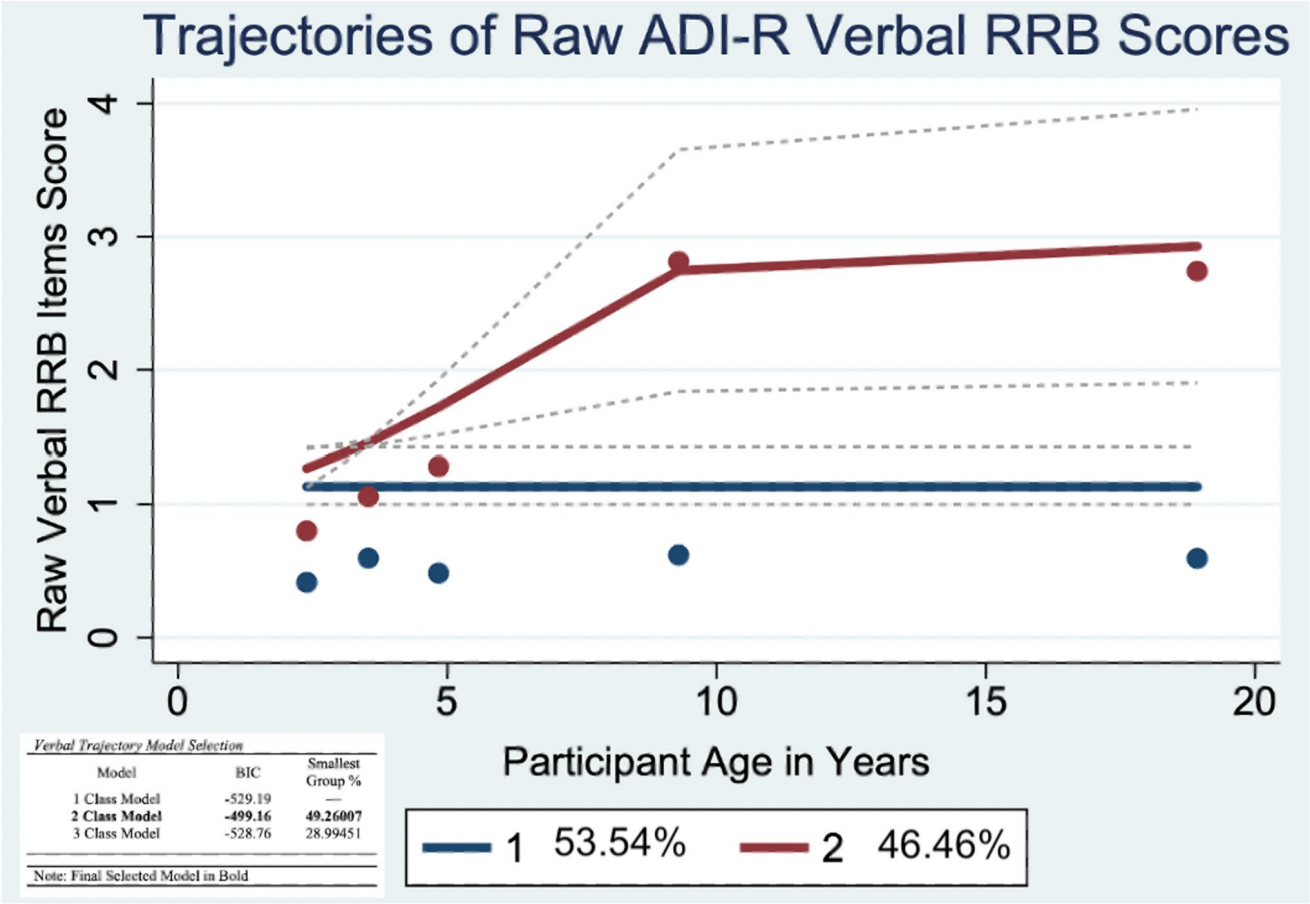
Trajectories of Raw ADI-R Verbal Restricted Repetitive Behavior (RRB) Scores

To examine trajectories of current ADI-R RRB subtype scores, traj plugin in Stata 16 was used to identify group-based trajectories. Group-based trajectory modeling estimates developmental trajectories via maximum likelihood estimation (i.e., full maximum likelihood [FIML]); missing data are handled by estimating the model using all available information. The best fitting model (linear, quadratic, etc.) and number of trajectory groups were determined using Bayesian Information Criteria (BIC). Five time points of ADI-R interviews (at ages 2, 3, 5, 9, and 19) were used as independent variables to analyze the latent grouping of individuals in their RRB trajectories. Unconditional 2, 3, and 4 class models were compared using Bayesian Information Criterion (BIC) and the smallest group membership percentage (Figs. 1, 2, 3). After classes were determined, higher order effects were tested to establish whether cubic, quadratic, linear, or intercept modeling best explained variation over time. To aid in model selection, the average posterior probabilities were evaluated to determine adequate model fit (above 0.70; Nagin et al., 2018). One-way ANOVA and chi-square analyses were used to compare the composition of the RSM (Table 2), IS (Table 3), and Verbal (Table 4) trajectory classes for gender, race, recruitment site, caregiver education, ADOS CSS scores and IQ.

**Table 2:** ADI RSM and IS factor loadings from early childhood to early adulthood

Factor	ADI-R Item	Age 2	Age 3	Age 5	Age 9	Age 19
RSM	*Repetitive Use of Objects*	.81	.74	.72	.73	.64
	*Unusual Sensory Interests*	.67	.81	.75	.70	.60
	*Hand and Finger Mannerisms*	.55	.60	.87	.73	.63
	*Other Complex Mannerisms*	.49	.70	.75	72	.51
IS	*Resistance to Trivial Changes in Environments*	.91	.77	1.00	.78	.59
	*Difficulties with Changes in Routine*	.80	.56	.67	.75	.38
	*Compulsions and Rituals*	.62	.30	.62	.47	.09

**Table 3:** Verbal, IS, and RSM factor loadings at ages 9 and 19

Factor	RRB item	Factor Loading	
		Age 9	Age 19
RSM	*Repetitive use of objects*	.73	.57
	*Unusual sensory interests*	.62	.61
	*Hand and finger mannerisms*	.71	.68
	*Other complex mannerisms*	.63	.51
IS	*Resistance to trivial changes in environments*	.53	.27
	*Difficulties with changes in routine*	.01	.27
	*Compulsions and rituals*	.31	.69
Verbal	*Stereotyped utterance and delayed echolalia*	.70	.83
	*Neologisms/idiosyncratic language*	.28	.26
	*Verbal rituals*	.66	.58
	*Circumscribed interests*	.33	.37
	*Unusual preoccupations*	.56	.40

The inclusion of a third Verbal RRBs factor significantly worsened CFA model fit at age 9 (CFI = 0.719, TLI = 0.650, RMSEA = 0.096) and age 19 (CFI = 0.724, TLI = 0.657, RMSEA = 0.093). Interestingly, IS item factor loadings at age 19 improved when the Verbal RRBs factor was included, though we did not see a similar trend for IS items at age 9. Though clinically verbal symptoms of RRBs are a meaningful component of the behavioral presentation of ASD, these results suggest that inter-individual variability on these items in the current sample is too large for the latent structure of these symptoms to be meaningfully explained by a single underlying construct

**Table 4:** Descriptive Characteristics by RSM, IS, and Verbal Trajectory Groups

	Repetitive Sensorimotor (RSM)								
	Total	Low-Decreasing		Moderate-Decreasing		High-Fluctuating			
	N = 193	n = 47	m SD	n = 98	m SD	n = 48	m SD	*X*^*2*^ (df,N)	
	*n*(%)								
Gender									
Male	167(86.53)	44	–	95	–	46	–	11.14 (2,193)	0.87
Female	26 (13.47)	1	–	3	–	1	–		
Ethnicity									
Non-Hispanic/Latino	185 (97.4)	105	–	108	–	29	–	1.46 (2, 193)	0.92
Hispanic/Latino	5 (2.6)	4	–	4	–	0	–		
White	135(69.94)	34	–	74	–	27	–	5.85(2, 193)	0.053
Black	54 (27.97)	12	–	24	–	18	–		
Race									
Asian	3 (1.5)	1	–	0	–	2	–		
American Indian	0 (0)	0	–	0	–	0	–		
Biracial	1 (.51)	0	–	0	–	1	–		
North Carolina	103(53.36)	27	–	49	–	27	–	6.61 (4,193)	0.15
Recruitment Site									
Illinois	75 (38.34)	15	–	39	–	21	–		
Michigan	15 (8.29)	5	–	10	–	0	–		
Caregiver Education									
< Four-year degree	151 (78.23)	35	–	81	–	35	–	2.31 (2, 193)	0.31
> Four-year degree	42 (21.76)	12	–	17	–	13	–		

								*t*	df,N 2, 175	*p*
Childhood IQ										
Verbal		43	44.47 (20.89)	87	34.74 (20.31)	48	23.25 (13.57)	14.44		< .001
Nonverbal		43	78.37 (19.75)	87	65.78 (19.02)	27	55.52 (20.86)	15.27	2, 175	< .001
Adulthood IQ										
Verbal		26	88.35 (35.37)	51	56.53 (44.29)	26	27.08 (31.59)	15.82	2, 100	< .001
Nonverbal		37	92 (33.4)	59	59.53 (39.78)	14	31.12 (25.73)	18.48	2, 93	< .001
ADOS CSS Scores										
Childhood		38	6.42 (2.62)	86	7.49 (2.43)	48	8.81 (1.32)	12.54	2, 169	< .001
Adulthood		24	4.83 (2.55)	54	5.3 (2.12)	17	4 (2.37)	2.12	2, 92	0.125
ADOS CSS Scores SA										
Childhood		38	7.11 (2.53)	86	7.84 (2.52)	48	9 (1.35)	7.88	2, 169	< .001
Adulthood		25	5.6 (2.47)	51	5.57 (2.29)	20	4.55 (2.48)	1.48	2, 93	0.232

	Incistence on Sameness (IS)						
	Total	Low-Increasing		High-Decreasing			
	N = 193	n = 162	m SD	n = 31	m SD	*X*^*2*^ (df,N)	
	*n*(%)						
Gender							
Male	167 (86.53)	140	–	27	–	0.01 (1,193)	0.91
Female	26 (13.47)	22	–	4	–		
Ethnicity							
Non-Hispanic/Latino	185 (97.4)	156	–	29	–	2.10 (1, 193)	0.14
Hispanic/Latino	5 (2.6)	3	–	2	–		
White	135 (69.94)	112	–	23	–	2.01 (1, 193)	0.36
Black	54 (27.97)	47	–	7	–		
Race							
Asian	3 (1.5)	2	–	1	–		
American Indian	0 (0)	0	–	0	–		
Biracial	1 (.51)	1	–	0	–		
North Carolina	103 (53.36)	84	–	19	–	3.49 (2,193)	0.17
Recruitment Site							
Illinois	75 (38.34)	63	–	12	–		
Michigan	15 (8.29)	15	–	0	–		
Caregiver Education							
< Four-year degree	151 (78.23)	127	–	24	–	0.014 (1, 193)	0.9
> Four-year degree	42 (21.76)	35	–	7	–		

						*t*	df,N	*p*
Childhood IQ								
Verbal		147	33.56 19.67	31	36 23.17	− 0.6	1, 175	0.545
Nonverbal		147	66.65 20.6	31	63.23 24.2	0.81	1, 175	0.416
Adulthood IQ								
Verbal		88	56.66 44.16	15	59.87 48.82	− 0.25	1, 100	0.798
Nonverbal		81	59.86 41.3	15	62.33 40.75	− 0.21	1, 93	0.832
ADOS CSS Scores								
Childhood		143	7.64 2.33	29	7.55 2.6	0.17	1, 170	0.862
Adulthood		83	4.93 2.32	12	5.08 2.31	− 0.21	1, 93	0.829
ADOS CSS Scores SA								
Childhood		143	8.07 2.3	29	7.66 2.59	0.86	1, 169	0.388
Adulthood		84	5.32 2.41	12	5.67 2.35	− 0.46	1, 93	0.642

	Verbal Restricted, Repetitive Behaviors (Verbal RRBs)						
	Total	Low-Stable		High-Increasing			
	N = 127	n = 68	m SD	n = 59	m SD	*X*^*2*^ (df,N)	
	*n*(%)						
Gender							
Male	110 (86.61)	60	–	50	–	.33 (1,127)	0.56
Female	17 (13.38)	8	–	9	–		
Ethnicity							
Non-Hispanic/Latino	122 (96.82)	66	–	56	–	1.31 (1, 127)	0.25
Hispanic/Latino	4 (3.18)	1	–	3	–		
White							
Black	96 (75.59)	48	–	48	–	3.71 (1, 127)	0.15
Race	38 (23.62)	20	–	10	–		
Asian	1 (0.7)	1	–	1	–		
American Indian	0 (0)	0	–	0	–		
Biracial	0 (0)	0	–	0	–		
North Carolina							
Recruitment Site	62(48.81)	35	–	27	–	.41 (2,127)	0.81
Illinois	50 (38.58)	26	–	24	–		
Michigan	15 (12.59)	7	–	8	–		
Caregiver Education							
< Four-year degree	102 (80.31)	51	–	51	–	2.61 (1, 127)	0.105
> Four-year degree	25 (19.68)	17	–	8	–		

						*t*	df,N	*p*
Childhood IQ								
Verbal		60	43.27 (21.62)	51	38.8 (19.95)	1.12	1, 109	0.26
Nonverbal		60	75.23 (19.48)	51	71.24 (16.79)	1.14	1, 109	0.25
Adulthood IQ								
Verbal		40	85.75 (32.83)	39	70.74 (39.53)	1.83	1, 77	0.07
Nonverbal		33	90.15 (30.04)	35	71.37 (34.88)	2.37	1, 66	0.02
ADOS CSS Scores								
Childhood		55	6.56 (2.64)	51	7.29 (2.48)	− 1.46	1, 104	0.14
Adulthood		38	5.11 (2.44)	39	5.31 (2.08)	− 0.39	1, 75	0.69
ADOS CSS Scores SA								
Childhood		55	7 (2.63)	51	7.57 (2.45)	− 1.14	1, 104	0.25
Adulthood		37	5.57 (2.4)	37	5.49 (2.21)	0.15	1, 72	0.88

To assess potential variation in the trajectories of specific types of RRBs, RSM score trajectories, which included all ADI-R C-section items assessing repetitive sensorimotor behaviors, IS score trajectories, which included all ADI-R C-section items assessing insistence on sameness behaviors, and verbal RRBs trajectories, which included all ADI-R C-section items section items at least partially reliant on verbal ability, were separately calculated. The ADI-R items included in each of the three symptom domain trajectories are listed in Table 1.

To account for the inclusion of communication symptoms in the RRB domain of DSM-5 ASD diagnostic criteria (APA, 2014), we also calculated totals for RSM trajectories that included raw item scores for stereotyped utterances and delayed echolalia, as well as totals for verbal trajectories that included raw item scores for stereotyped utterances, delayed echolalia and neologisms/idiosyncratic language. The inclusion of these items in the RSM and verbal trajectories did not demonstrably change the trajectory group membership percentages or patterns of growth (see Figure S3). Thus, we proceeded with the original domains based on the ADI-R algorithms and prior factor analyses, rather than DSM-5 diagnostic criteria.

#### Aim 3: Investigate Rates of Change in RSM, IS, and Verbal Trajectory Subgroups

Multilevel modeling via the mixed procedure in Stata was used to investigate differences in the intercepts and slopes (i.e., rates of change) of the RSM, IS, and verbal trajectory subgroups, respectively. A multilevel modeling approach was selected for two reasons. First, because participants were nested across time points, multilevel modeling allowed us to account for the non-independence of assessment observations. Second, this approach enabled us to evaluate the influence of the inclusion of a random slope on trajectory estimates, and to allow for the possibility of differing slopes before and after late childhood. Given prior findings that core ASD symptomology may peak in late childhood (Bal et al., 2019; Gotham et al., 2012; Richler et al., 2010), we leveraged a piecewise approach to assess differences in the slopes of RRB subtypes before and after our last ADI-R data point in childhood, age 9. Missing data were handled by estimating models using all available information (i.e., FIML). The models estimated included random slopes for trajectory group and fixed effects for age, trajectory group, slope after age 9, as well as age x trajectory group, age x slope, slope x trajectory group, and age x trajectory groups x slope interaction terms. All models were grand mean centered for age. Descriptive variables identified by one-way ANOVA and chi-square analyses as differing significantly between trajectory groups were included in the multilevel models as covariates. To account for multiple comparisons, Bonferroni corrections were used in interpreting all descriptive analyses.

## Results

### Aim 1: ADI-R RRB Domain Factor Structure

#### Two Factor Model (RSM & IS) at Age 19

A CFA model including age 19 IS and RSM items only was tested to examine whether the two-factor model derived in prior analyses of ADI-R data from this sample in childhood remained consistent at age 19. This model adequately fit the data (CFI = 0.964, TLI = 0.943, RMSEA = 0.043). Factor loadings are reported in Table 1. Item loadings across the RSM factor were high, ranging from 0.51 to 0.64. One item in the IS factor, *Compulsions and Rituals* had a notably poor loading of 0.09 at age 19. Excluding *Compulsions and Rituals*, item loadings on the IS factor ranged from 0.38 to 0.59.

#### Three Factor Models (RSM & IS, & Verbal RRBs) at Ages 9 and 19

Two additional CFA models examined the effects of including a third factor, Verbal RRBs, on model fit at age 19 as well as age 9, as language fluency from ages 9 to 19 was quite stable in this sample (Lord et al., 2004). The inclusion of a third factor, Verbal RRBs, in the second CFA model considerably worsened model fit both at age 9 (CFI = 0.719, TLI = 0.650, RMSEA = 0.096) and at age 19 (CFI = 0.724, TLI = 0.657, RMSEA = 0.093). Thus, only the previously-identified IS and RSM factors were retained. Factor loadings for the age 9 and 19 CFA models including a Verbal RRBs factor are reported in Table S1.

### Restricted Sensory Motor (RSM) Behaviors

#### Aim 2: RSM Trajectories

The best fitting model was a three-group model (Fig. 1). The first trajectory group, Low-Decreasing, consisted of 28.2% of the sample. The second trajectory group, Moderate-Decreasing, consisted of 44.4% of the sample. Both the Low- and Moderate-Decreasing trajectories were best described by linear modeling. The third RSM trajectory group, High-Fluctuating, comprised 27.3% of the sample and was best described by quadratic modeling. Compared to participants in the Low- and Moderate-Decreasing trajectory groups, those in the High-Fluctuating RSM group had significantly lower childhood verbal IQ (F = 14.44; p < 0.001); childhood nonverbal IQ (F = 15.27; p < 0.001); adult verbal IQ (F = 15.82; p < 0.001); adult nonverbal IQ (F = 18.84; p < 0.001); and higher childhood ADOS overall CSS scores (F = 12.54; p = 0.001); as well as higher childhood ADOS SA CSS scores (F = 7.88; p = 0.001; Table 2). There were no significant differences in race nor gender between each of the RSM trajectory groups (Table S2).

#### Aim 3: Rate of Change in RSM Trajectories

Multilevel modeling was used to compare rates of change in the three RSM trajectory groups from ages 2–19. The inclusion of a main effect and interaction effects for slope did not significantly contribute to the RSM model and were thus dropped from the final model. There was also no significant effect of age in the RSM model (*p* = 0.294). However, there was a significant effect of group membership, with individuals in both the Moderate-Decreasing and High-Fluctuating groups having significantly higher intercepts (i.e., RSM raw scores) than individuals in the Low-Decreasing group (both *p* < 0.001). There was also a significant age x trajectory group interaction, indicating that inclusion in the Moderate-Decreasing group was associated with a significantly steeper decline in RSM raw scores between ages 2–19 than inclusion in the other two trajectory groups (*p* = 0.003). The inclusion of a trajectory group random-level slope significantly improved model fit (*p* < 0.05), indicating rates of change in the three RRB Total trajectory groups significantly differed from one another. Finally, childhood ADOS SA CSS scores significantly contributed to the model (*p* = 0.001), though recruitment site (*p* = 0.61) and childhood nonverbal IQ (*p* = 0.24) did not.

### Insistence on Sameness (IS) Behaviors

#### Aim 2: IS Trajectories

A two-group model best fit the raw IS score trajectories. The first group, Low-Increasing, consisted of 18.5% of the sample; the second trajectory group, High-Decreasing, consisted of 81.5% of the sample. The Low-Increasing group was best described by cubic modeling, and the High-Decreasing group was best described by quadratic modeling. There were no significant differences between descriptive characteristics of the two IS trajectory groups (Table 3, Table S3).

##### Aim 3: Rate of Change in IS Trajectories

There was a significant effect of age in the IS model (*p* < 0.001) and a significant effect of group membership, indicating participants in the High-Increasing trajectory group had significantly higher IS raw scores than participants in the Low-Decreasing group (*p* < 0.001). Age x trajectory group, trajectory group x slope, and age x trajectory group x slope interaction terms were not significant. However, there was a significant slope x age interaction, indicating rates of change for both the High-Increasing and the Low-Decreasing before age 9 were significantly different from the rates of change in both groups after age 9 (*p* < 0.001). In the Low-Decreasing group, IS scores were elevated at age 2 and increased slightly through age 9, then significantly declined from ages 9–19. In contrast, the High-Increasing group had low IS scores at age 2 and experienced a larger increase in IS scores from ages 2–9 than the Low-Decreasing group, followed by a plateau in IS scores from ages 9–19. The inclusion of random trajectory group level slopes significantly improved model fit (*p* < 0.05).

### Verbal Restricted and Repetitive Behaviors

#### Aim 2: Verbal RRBs Trajectories

A two-group model was determined to be the best fit for the verbal RRB trajectories. The first group, Low-Stable, consisted of 53.54% of the sample, and the second trajectory group, High-Increasing, consisted of 46.46% of the sample. The Low-Stable group was best described by intercept modeling, while the High-Increasing group was best described by quadratic modeling. The Low-Stable group had a significantly higher adulthood non-verbal IQ than the High-Increasing group (t = 2.37; p = 0.02; Table 4). There were no significant differences in race nor gender between each of the Verbal RRB trajectory groups (Table S4).

#### Aim 3: Rate of Change in Verbal RRB Trajectories

There was no significant effect of age (*p* = 0.79) but a significant effect of group membership (*p* < 0.001) in the verbal model. There were also significant age x trajectory group (*p* < 0.001) and age x trajectory group x slope (*p* < 0.001) interaction terms. The inclusion of random trajectory group level slopes also significantly improved model fit (*p* < 0.05). Participants in the High-Increasing trajectory group had significantly higher verbal RRB raw scores (i.e., intercepts) than participants in the Low-Stable group. Verbal RRB raw scores increased in both trajectory groups over time, however, the scores of individuals in the High-Increasing group increased at a faster rate from ages 2–9. For both trajectory groups, verbal RRB scores did not significantly increase or decrease from ages 9–19 (Table 5).

**Table 5:** Rates of Growth in RSM, IS, and Verbal RRB Trajectories

Fixed Effects	RSM			IS			Verbal		
	n = 193		95% CI	n = 193		95% CI	n = 127		95% CI
Participant Age	− 0.02 (.02)		[− 0.06, 0.02]	0.18 (.03)		[0.11, 0.25]	− 0.01 (.04)		[− 0.11, 0.10]
Trajectory Group	Moderate− Decreasing	2.34 (.18)**	[1.98, 2.70]	High− Decreasing	2.38 (.34)**	[1.73, 3.03]	High− Increasing	1.91 (.23)***	[1.39, 2.39]
	High− Fluctuating	4.91 (.22)***	[4.46, 5.33]						
Age x Trajectory Group	Moderate− Decreasing	− 0.08 (.02)*	[− 0.14. − 0.02]	High− Decreasing	− 0.09 (.08)	[− 0.26, 0.06]	High− Increasing	0.30 (.05)***	[0.28, 0.49]
	High− Fluctuating	− 0.02 (.03)	[− 0.09, 0.04]						
Age x Slope in Adulthood	–		–	− 0.19 (.04)***		[− 0.28, − 0.11]	0.73 (.20)***		[0.19, 0.42]
Trajectory Group x Slope in Adulthood	–		–	High− Decreasing	0.29 (.60)	[− 0.48, 0.52]	High− Increasing	1.23 (.28)***	[0.68, 1.78]
Age x Trajectory Group x Slope in Adulthood	–		–	High− Decreasing	− 0.08 (.09)	[− 0.80, 1.32]	High− Increasing	− .36 (.06)***	[− 0.46, − 0.27]
Childhood Non-Verbal IQ	− 0.004 (.003)		[− 0.01, 0.002]	–		–	–		–
Childhood ADOS SA CSS	0.10 (.03)**		[0.04, 0.16]	− 0.01 (.02)		[− 0.06. 0.02]	0.02 (.02)		[− 0.14, 0.06]
Random effects									
Trajectory Group	0.01 (.02)*		[− 2.23, − 1.59]	0.10 (.04)*		[0.05, 0.34]	.13 (.05)*		[0.05, 0.28]

*p < .05, **p < .01, ***p ≤ *.*001

The reference groups for trajectory group and the age x trajectory interaction terms for the RSM, IS, and Verbal models are Low-Decreasing, Low-Increasing, and Low-Stable, respectively. Terms not included in the final models due to a lack of significant associations are indicated by dashed lines in the table. Independent t-tests and one-way ANOVAs were used to determine model covariates. For example, because participants in the High-Fluctuating RSM trajectory group had lower childhood non-verbal IQ and higher childhood ADOS SA scores than participants in the Moderate-Decreasing and Low-Decreasing trajectory groups, both covariates were included in the RSM model

## Discussion

This study demonstrates that individuals with ASD experience different trajectories of RRB subtypes—specifically, restricted sensorimotor, insistence on sameness, and verbal restricted repetitive behaviors—from early childhood to young adulthood. It also found evidence that the two-factor structure of ADI-R RRB domain items remains consistent into early adulthood, however, the fit of this two-factor structure at age 19 was notably poorer than at earlier ages. The inclusion of a third factor, Verbal RRBs, significantly worsened CFA model fit for at both age 9 and age 19 and was ultimately omitted. Our results suggest that inter-individual variability on the items assessing verbal RRBs on the ADI-R may be too large for the latent structure of these symptoms to be meaningfully explained by a single underlying construct (at least in the current, relatively small, sample—see below). However, as reflected by the consolidation of RRB and communication symptoms of autism in DSM-V diagnostic criteria (APA, 2014), verbal symptoms of RRBs are a clinically meaningful component of the behavioral presentation of ASD. With this in mind, we chose to retain our verbal RRB trajectory and rate of change analyses with the hope that this information will be clinically valuable to the conceptualization and treatment of RRBs in verbally fluent autistic individuals.

RSM and IS factors previously identified using childhood ADI data appeared consistent with scores from this sample in early adulthood. Notably, age 19 factor loadings for IS were lower than RSM, with the item *Compulsions and Rituals* having an especially low factor loading of 0.09 at age 19. This is somewhat mirrored by our IS trajectories findings, as almost 20% of our sample experienced a significant decrease in IS behaviors from late childhood to early adulthood (Fig. 2). It is possible that this variation in IS factor structure is reflective of the developmental changes (i.e., declines) autistic individuals may experience in their IS behaviors after childhood.

Inclusion of a verbal factor considerably worsened CFA model fit at both age 9 and age 19, though loadings for the Verbal, IS, and RSM factors were relatively high (Table 3). Some have argued that the CFI, TLI, and RMSEA cutoffs for goodness of fit statistics are overly exclusive in small samples (Hu & Bentler, 1999). It is possible that some of the issues observed here with factor loadings and model fit for the verbal RRBs factor are due to the relatively small sample size of the present study. Given that many of the participants in the current sample did not have fluent verbal abilities by age 5, as indicated by scores greater than 0 on the ADI-R “Overall Level of Language” item, and/or that caregivers of many participants did not endorse many verbal symptoms prior to age 9 (Table 6) we were unable to explore the utility of including a verbal factor at earlier ages. As described below, the relatively late emergence of verbal fluency seen in this sample may be quite different from samples of young children with autism identified today. With this in mind, and given prior evidence of verbal factors in other analyses of RRBs (Hiruma et al., 2021; Lam et al., 2008), future work should continue to explore the factor structure RRBs in relation to verbal ability.

**Table 6:** ADI-R item endorsement at ages 2, 3, 5, 9, and 19

ADI-R item	Percentage of Participants with Item Endorsed				
	Age 2 (%)	Age 3 (%)	Age 5 (%)	Age 9 (%)	Age 19 (%)
Verbal rituals	3.27	12.37	14.60	19.47	17.76
Unusual preoccupations	34.11	39. 25	31.88	28.42	20.77
Circumscribed interests	0	0	5.07	39.47	44.15
Stereotyped utterances and delayed echolalia	10.74	26.34	36.23	51.05	41.44
Neologisms/idiosyncratic language	2.34	13.44	10.22	14.21	7.24

Despite this null finding, we feel strongly that it is meaningful to report on developmental trajectories of verbal symptoms here given the clinical significance of these behaviors to the presentation of RRBs in verbally fluent individuals with autism. Arguably, RSM and some IS behaviors are more likely to be readily apparent in autistic individuals of varying verbal abilities compared to verbal behaviors such as circumscribed interests and unusual preoccupations, which may only be noticeable in autistic individuals who can describe their interests and preoccupations at length.

On average, symptoms of all three RRB subtypes significantly declined or plateaued by early adulthood. We identified three patterns of RSM trajectories: Low-Decreasing, Moderate-Decreasing, and High-Fluctuating. The Low- and Moderate-Decreasing RSM groups had differing intercepts but similar declines across development. In contrast, the High-Fluctuating group experienced an increase in RSM symptoms from ages 2–9, followed by a decrease from ages 9–19. IS trajectories followed High-Decreasing or Low-Increasing patterns. Finally, verbal RRBs followed High-Increasing or Low-Stable trajectories. Notably, there were no differences amongst RSM, IS, or Verbal trajectory subgroups by race/ethnicity, gender, or recruitment site (Tables S1–S3). IQ and ADOS CSS scores did not significantly differ between the IS and Verbal trajectory groups, and thus were not included in the corresponding multilevel models. In contrast, IQ scores were significantly lower and ADOS CSS scores were significantly higher amongst participants in the High-Fluctuating RSM group than the other trajectory groups. IQ scores did not significantly contribute to the RSM multilevel model, however, ADOS CSS Social Affect (SA) scores did significantly contribute to the model, suggesting higher total autism symptom severity, not just higher RRB symptom severity was associated with more variability in RSM trajectories across development.

By longitudinally characterizing change and stability in RRB subtypes from ages 2–19, this paper adds to previous work in this sample (Richler et al., 2010) as well as work conducted in other samples (Bishop et al., 2013; Uljarević et al., 2021) that found differing profiles of RRB symptoms in autistic individuals both within and across age groups. This study also corroborates findings from other longitudinal samples that, on average, core ASD symptomology decreases with increasing age (Seltzer et al., 2004; Taylor & Seltzer, 2010). We do not want to suggest that the declines in RSM and IS behaviors described here necessarily indicate a decline in the impairments associated with ASD in early adulthood. In describing their findings of improvements in social communication behaviors with increasing age in a prior analysis of this sample, Bal et al. (2019) astutely noted, “while it is tempting to interpret symptom changes as representative of a decrease in autism severity, it is perhaps more appropriate to consider improvements as evidence of ongoing development” (p. 96). In other words, ASD is a lifespan developmental condition, and as such, core symptomology of ASD, including RRBs, varies meaningfully across the life course. The RRB domain of the ADI-R appears to be sensitive to such developmental changes while still maintaining a consistent two-factor internal structure, though notably, the fit of this two-factor structure, particularly the IS factor, worsened in early adulthood. Given the increasing numbers of adolescents and adults receiving autism diagnoses for the first time, additional work should continue to explore the utility of the ADI-R as a diagnostic instrument for older individuals as well as young children.

For participants in the Low-Decreasing (28.2%) and Moderate-Decreasing (44.4%) RSM trajectory groups—almost three-quarters of the current sample—RSM behaviors were most pronounced in early childhood between ages two and three, then declined through early adulthood (Fig. 1). In contrast, participants in the High-Fluctuating group (27.3%) had more RSM symptoms in early childhood than participants in the other trajectory groups and experienced an increase in RSM symptoms from ages 2–9. However, the High-Fluctuating group’s RSM symptoms steadily declined in late childhood and adolescence such that by age 19, participants in the High-Fluctuating group had similar levels of RSM symptoms as in early childhood.

Surprisingly, RSM behaviors were the only subgroup of RRBs examined here where patterns of change over time were associated with other phenotypic characteristics. This somewhat contrasts with prior work on social communication symptoms in this sample (Bal et al., 2019) and other longitudinal samples of autism (Shattuck et al., 2007), which found trajectories of social communication symptoms significantly varied based on childhood non-verbal IQ. Inclusion in the High-Fluctuating RSM trajectory was associated with lower IQ and more pronounced social communication impairments in childhood and adulthood (Table 3). These findings suggest that, in contrast to social communication impairments, RRBs are distinct from IQ.

In contrast to the positive linear growth seen prior to age 9, IS behaviors decreased from 9–19 in the High-Decreasing group and plateaued during this same period in the Low-Increasing group (Fig. 2). In part, insistence on sameness behaviors may increase from early to late childhood due to natural developmental changes. For example, a two-year-old unhappy with a small change in the placement of a living room chair may not be able to effectively communicate their distress or physically move the chair back to its original “correct” placement. However, a 9-year-old could do both, increasing the likelihood that their caregiver would endorse IS symptoms during the ADI-R. In this scenario, the child’s core IS symptomology may not necessarily change from early to late childhood, but the ability to make caregivers aware of these symptoms may increase markedly.

In addition to replicating and extending prior findings on RSM and IS symptom profiles in childhood and adulthood (Bishop et al., 2013; Evans et al., 2017; Guthrie et al., 2013; Richler et al., 2010), this study described distinct developmental trajectories of verbal RRBs. Only participants that received a score of 0 at one or more timepoints on the ADI-R item “Overall Level of Language,”—indicating daily, functional use of language involving phrases of three or more words—were included in the verbal trajectories. Within this subsample, we identified two verbal RRB trajectory groups, Low-Stable (53.54%) and High-Increasing (46.46%). The rate of growth in verbal RRBs for the High-Increasing group significantly differed before and after age 9. The Low-Stable group displayed few to no verbal RRBs from ages 2–19; in contrast, the High-Increasing group experienced a sharp increase in verbal RRBs between ages 2–9, at which point their verbal RRBs plateaued. On average, participants in the High-Increasing group had higher non-verbal IQs in adulthood than participants in the Low-Stable group, though there were no significant differences between the groups in childhood verbal or non-verbal IQ. Unsurprisingly, we did see significant IQ differences between the Low-Stable and High-Increasing groups when we included participants who had scores other than 0 on the ADI-R item “Overall Level of Language,” in the verbal trajectory analysis, as many of these individuals had IQ of less than 70 (Figure S1). Retaining only participants with relatively strong language skills in the verbal trajectories allowed us to more sensitively examine variation in this subset of symptoms. It is remarkable that even amongst autistic individuals with phrase speech or better, more than half had very few verbal symptoms from early childhood through early adulthood. This may suggest that in contrast to RSM and IS behaviors, verbal symptoms of autism occur somewhat less frequently.

A recent study of RRBs in minimally verbal children with ASD receiving intervention services found verbal RRBs such as echolalia, scripting and repetitive language and sounds increased during intervention (Harrop et al., 2021). Harrop et al. attributed this increase in verbal RRBs to increases in overall language production in response to intervention. For autistic individuals with fluent speech, certain RRBs such as unusual preoccupations and circumscribed interests may be more apparent to caregivers, particularly if the autistic individual in question enjoys speaking about their restricted interests at length. In short, verbal RRBs appear to emerge in step with language fluency and stabilize as language fluency stabilizes, suggesting childhood surges in verbal RRBs could be indicative of increasing language fluency.

The DSM-5 collapsed communication symptoms such as idiosyncratic phrases and echolalia into the RRB diagnostic criteria (APA, 2014). To reflect these DSM-5 inclusions in the characterization of RRBs, in addition to the RRB subtype totals summarized in Table 1, we also calculated RSM trajectory totals including raw item scores for stereotyped utterances and delayed echolalia and verbal RRB trajectory totals including raw item scores for stereotyped utterances, delayed echolalia and neologisms/idiosyncratic language. The inclusion of these additional items reflecting DSM-5 criteria did not change the number of RSM and Verbal RRB trajectory groups nor the patterns of growth of each trajectory groups for RSM and Verbal RRBs (Figures S2 and S3). In other words, the inclusion of these additional symptoms in the DSM-5 criteria for the RRB symptom domain does not appear to alter the conceptual understanding of RRBs. Future work in large samples comparing the relative fit of RRB factor structures based on DSM-5 and existing ADI-R RRB criteria could shed further light on this issue.

### Limitations

This sample, followed longitudinally since initial evaluation in the early 1990’s, is comprised of a unique group of adults with ASD. Participants received an ASD diagnosis approximately 30 years ago, during an era when early diagnoses of autism were relatively uncommon. Thus, this sample may not be representative of people diagnosed with autism today. The emergence of fluent speech was quite delayed in our sample, which may have contributed to the early identification of these participants at a time when autism was much less widely known. Unfortunately, this delayed language development prevented us from examining the fit of a verbal RRBs factor in the ADI-R factor structure prior to age 9. Further, speech delay may be less common in children with ASD diagnosed today. African American participants and participants with low caregiver education in this cohort were more likely to have missing data. Having relatively few females in our sample prevented examinations of potential gender differences in RRBs; such differences have been identified in prior work (Frazier et al., 2014).

Though the CFA models reported here were fit using all available ADI data from this sample at ages 9 and 19, attrition and our relatively small sample size, particularly in the context of factor analyses, may have limited our ability to examine the latent structure of RSM, IS, and verbal behaviors adequately in late childhood early adulthood. Future work should continue to examine the factor structure of RRBs, particularly in large-scale samples of adults with autism. It is possible that the underlying structure of RRBs changes in adulthood. Given that the ADI-R was designed to assess symptoms of autism in children, such changes may not be adequately captured by this instrument.

### Future Directions

Future studies should continue to examine patterns of RRB development into middle and later adulthood. Though research examining ASD symptoms and the experiences of autistic individuals and their families in early adulthood has increased in recent years, our understanding of ASD in middle and later adulthood remains limited at best (Piven & Rabins, 2011). Prospectively monitoring how factors like gender and types of interventions and supports received affect long-term trajectories of core ASD symptoms also remains important.

Understanding change and stability in core ASD symptomology throughout the life course is relevant to clinical practice. The current study complements prior work in this sample characterizing stability and change in social communication symptoms of ASD from early childhood to adulthood (Bal et al., 2019). In treatment planning, understanding how a patient’s RRB symptomology may change over time and how this relates (or does not relate) to change in other important phenotypic characteristics, including IQ and social communication behaviors, can help clinicians and family members prioritize potential treatment targets. Further, developmental trajectories of RRBs may help clinicians appropriately apply ASD diagnostic criteria across the lifespan, as a young adult with autism may present differences in RRBs in symptom type, number, and intensity than a young child. For example, verbal RRBs may be more apparent after late childhood, while IS behaviors may diminish during this same period

## Conclusions

As increasing numbers of autistic individuals enter adulthood, understanding developmental changes in the presentation of ASD becomes increasingly important. The present study provides insight into how RRBs may shift from early childhood into adulthood, which could inform when clinicians should monitor for increases in RRB symptoms across the life course. Importantly, the IS and RSM factors identified in analyses of ADI data from childhood also adequately fit ADI data from age 19, though the fit of the IS factor decreased with increasing age. In the current sample, RSM behaviors declined from 2–19, with slightly less than one-third (27.3%) of participants experiencing an increase in RSM behaviors from 2–9, followed by a decrease from 9–19. IS behaviors increased from ages 2–9, then declined or plateaued from 2–19, and Verbal RRBs increased from 2–9, then plateaued from 2–19—though 38.8% of the sample displayed few or no Verbal RRBs across development. Higher CSS SA scores were associated with more RSM symptoms across development. However, CSS SA scores were not related to the IS and Verbal trajectory groups, and non-verbal IQs from early childhood were not related to change in the RSM, IS, or Verbal trajectories. Future work should assess change and stability in RRB symptoms in autistic individuals through middle and later adulthood and examine relationships between RRB trajectories and other adult outcomes such as employment, living status, and quality of life.

## Supplementary Information

Below is the link to the electronic supplementary material.

Supplementary file1 (DOCX 514 kb)
