# Supplementary material for: Development of Restricted and Repetitive Behaviors from 2–19: Stability and Change in Repetitive Sensorimotor, Insistence on Sameness, and Verbal Behaviors in a Longitudinal Study of Autism
Source: J Autism Dev Disord. 2024 May 14;55(7):2254–71. doi: 10.1007/s10803-024-06307-4 (PMC12167264; doi:10.1007/s10803-024-06307-4)
Supplement: Supplementary file 1 — Supplementary file1 (DOCX 514 kb) [file 10803_2024_6307_MOESM1_ESM.docx]

**Supplementary Tables and Figures**

Supplementary Tables

*Supplementary Table 1.* Descriptive Characteristics of RSM Trajectory Groups

*Supplementary Table 2.* Descriptive Characteristics of IS Trajectory Groups

*Supplementary Table 3.* Descriptive Characteristics of Verbal RRBs Trajectory Groups

Supplementary Figures

*Supplemental Figure 1.* Trajectories Of Raw Verbal RRB Scores Including Verbal And Non-verbal/minimally Participants.

*Supplemental Figure 2.* Trajectories Of Raw Verbal RRB Scores Including Additional Items: Stereotyped Utterances and Delayed Echolalia; and Neologisms/Idiosyncratic Language

*Supplemental Figure 3.* Trajectories Of Raw RSM Scores Including Additional Item: Stereotyped Utterances and Delayed Echolalia

**Table S1.** Descriptive Characteristics of RSM Trajectory Groups

**Table S2.** Descriptive Characteristics of IS Trajectory Groups

**Table S3.** Descriptive Characteristics of Verbal RRB Trajectory Groups

**Figure S1.** Trajectories Of Raw Verbal RRB Scores Including Verbal and Non/minimally-Verbal Participants


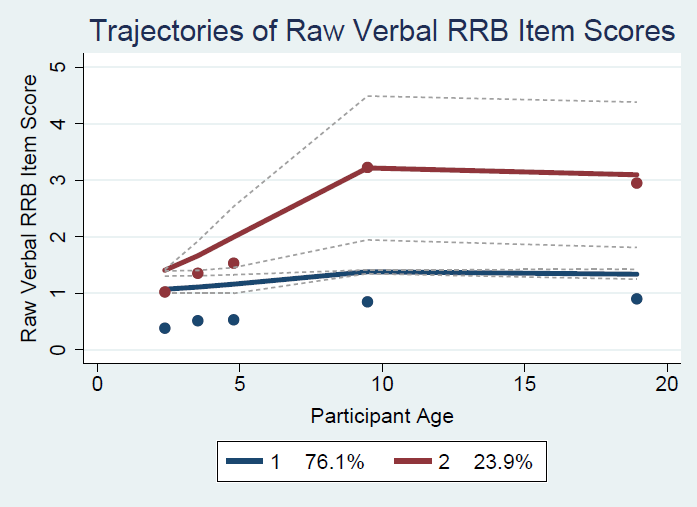


Group-based trajectory modeling was used to determine trajectories of raw verbal RRB Item scores for non/minimally verbal participants and verbal participants. A two-group model was determined to be the best fit. The first trajectory group consisted of 76.1% of the sample, and the second group, consisted of 23.9% of the sample. Both groups were best described by quadratic modeling. Growth patterns were similar between the models with and without the non/minimally verbal participants (Figure 3; Figure S1). However, trajectory group percentages differed, with a higher proportion of participants in the Low-Stable group in the model including non/minimally verbal. This shows that verbal ability may contribute to Verbal RRB presentation in people with autism.  ​

**Figure S2.** Trajectories Of Raw Verbal RRB Scores Including Additional Items: Stereotyped Utterances and Delayed Echolalia; and Neologisms/Idiosyncratic Language


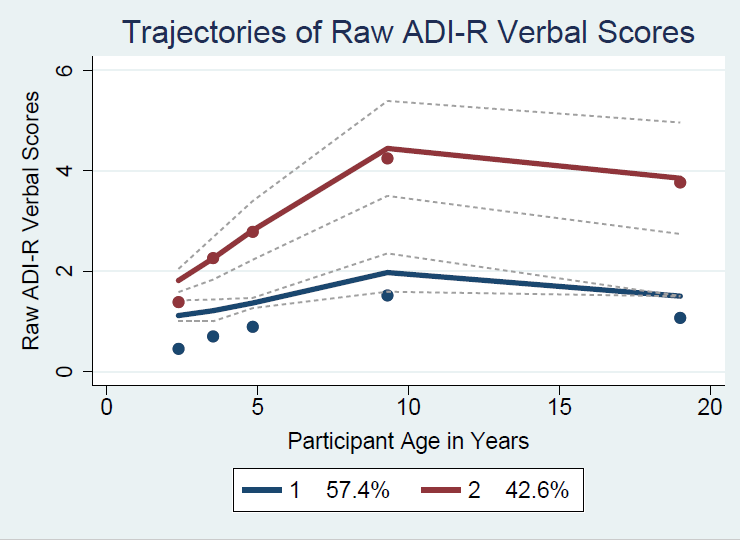


*Trajectories of Verbal RRBs with Additional Items.* Group-based trajectory modeling was used to determine trajectories of raw verbal RRB scores for non/minimally-verbal and verbal participants. These scores consisted of ADI-R RRB items contingent on verbal ability (Figure 1) with two additional items: Stereotyped Utterances and Delayed Echolalia; and Neologisms/Idiosyncratic Language. These additional items were added to reflect updates made to the DSM-5 (APA, 2014). A two-group model was determined to be the best fit. The first trajectory group consisted of 57.4% of the sample, and the second group, consisted of 42.6% of the sample. Both groups were best described by quadratic modeling. The inclusion of these items in the Verbal RRB domain did not demonstrably change the trajectory group membership percentages or patterns of growth.

**Figure S3.** Trajectories Of Raw RSM Scores Including Additional Item: Stereotyped Utterances and Delayed Echolalia


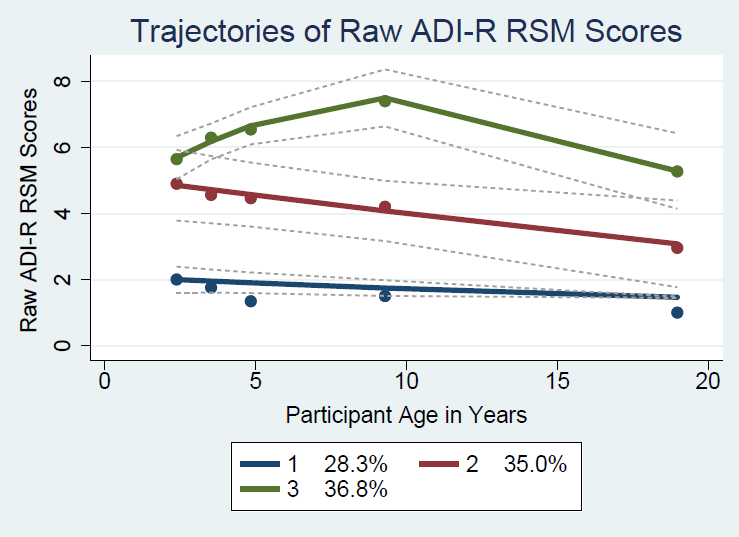


*Trajectories of RSM Behaviors with Additional Items.* Group-based trajectory modeling was used to determine trajectories of raw RSM scores for non/minimally-verbal and verbal participants. These scores consisted of ADI-R RSM items (Figure 1) with an additional item: Stereotyped Utterances and Delayed Echolalia. This additional item was added to reflect updates made to the DSM-5 (citation). A three-group model was determined to be the best fit. The first trajectory group consisted of 28.3% of the sample, the second group, consisted of 35.0% of the sample, and the last group consisted of 36.8% of the sample. The inclusion of these items in the RSM domain did not demonstrably change the trajectory group membership percentages or patterns of growth.
